# Structured caustic vector vortex optical field: manipulating optical angular momentum flux and polarization rotation

**DOI:** 10.1038/srep10628

**Published:** 2015-05-29

**Authors:** Rui-Pin Chen, Zhaozhong Chen, Khian-Hooi Chew, Pei-Gang Li, Zhongliang Yu, Jianping Ding, Sailing He

**Affiliations:** 1 Department of Physics, Zhejiang Sci-Tech University, Hangzhou, 310018, China; 2 National Laboratory of Solid State Microstructures and School of Physics, Nanjing University, Nanjing 210093, China; 3 Department of Physics, Faculty of Science, University of Malaya, Kuala Lumpur 50603, Malaysia; 4 Centre for Optical and Electromagnetic Research, Zhejiang University; Joint Research Center of Photonics of the Royal Institute of Technology (Sweden) and Zhejiang University (China)

## Abstract

A caustic vector vortex optical field is experimentally generated and demonstrated by a caustic-based approach. The desired caustic with arbitrary acceleration trajectories, as well as the structured states of polarization (SoP) and vortex orders located in different positions in the field cross-section, is generated by imposing the corresponding spatial phase function in a vector vortex optical field. Our study reveals that different spin and orbital angular momentum flux distributions (including opposite directions) in different positions in the cross-section of a caustic vector vortex optical field can be dynamically managed during propagation by intentionally choosing the initial polarization and vortex topological charges, as a result of the modulation of the caustic phase. We find that the SoP in the field cross-section rotates during propagation due to the existence of the vortex. The unique structured feature of the caustic vector vortex optical field opens the possibility of multi-manipulation of optical angular momentum fluxes and SoP, leading to more complex manipulation of the optical field scenarios. Thus this approach further expands the functionality of an optical system.

The phase, amplitude and polarization states are the fundamental parameters of an optical field^1^,^2^,^3^. An emerging vision for future advances in modern photonics is to explore the fundamental concepts of light coherently shaped in phase, amplitude and polarization^1^. Manipulating optical fields are especially required when light can be controlled by light itself, with novel properties emerging in connection with the optical beams having complex structures. Research on structured light and optical forces has cultivated a new, vigorous and distinctive interdisciplinary area, triggering strong interest and activity worldwide. Many of these new methods exploit the distinctive angular momentum, nodal architecture, and phase properties of beams with a complex wavefront structure^1^,^2^,^3^,^4^. Vortices have been intensively studied because of their intriguing properties and various applications such as stimulated emission depletion (STED) microscopy^5^, vortex solitons^6^ and trapping of particles^7^. Considerable interest in the orbital angular momentum of light beams arises from its potential use in a variety of applications in quantum information processing, atomic manipulation, micromanipulation and biosciences^7^,^8^,^9^,^10^,^11^. Singular optics such as phase vortices and polarization singularities has attracted immense attention and become important branches of the field^1^,^2^,^3^. Many works have been performed both theoretically and experimentally to understand the underlying physics and to explore the application of the singular characteristic of optical beams in linear and nonlinear optics^11^,^12^,^13^,^14^,^15^,^16^,^17^,^18^,^19^.

Recently, the study of vector optical fields, whose SoP are spatially variant in the field cross section, has attracted immense attention in linear and nonlinear optical realms due to their novel properties and potential applications^12^,^13^,^14^,^15^,^16^,^17^,^18^,^19^,^20^,^21^,^22^,^23^,^24^,^25^,^26^,^27^,^28^,^29^. Manipulation of polarization has attracted rapidly growing interest in their fundamental physics and applications. Various methods for generating vector beams have been proposed^22^,^23^,^24^,^25^,^26^,^27^,^28^,^29^,^30^. When the space-variant SoP in the field cross-section can span the entire surface of the Poincaré sphere, this kind of vector field is known as a Poincaré beam^20^. A higher-order Poincaré sphere representation of the higher-order SoPs in vector vortex beams has also been presented and demonstrated^21^. The corresponding experimental generation and properties of the standard and higher-order Poincaré beams have been reported^22^,^23^,^24^,^25^,^26^,^27^,^28^. The influence of the Gouy phase in vector-vortex beams due to the converging and diverging conical waves has been presented^31^. Recently, the complex fields with the arbitrary manipulation of the phase, amplitude and polarization (ellipticity and orientation) in the field cross-section have been reported^29^,^30^. A vector optical field can be generated from the output of novel lasers with specially designed or modified laser resonators^22^,^23^. In general, most methods are based on the wavefront reconstruction of the output beam from the traditional lasers, with the aid of specially designed optical elements^24^,^25^,^26^,^27^,^28^,^29^,^30^. In particular, a spatial light modulator (SLM) could provide the unique opportunity to flexibly design the arbitrary spatial (phase and amplitude) modulation patterns to generate the desired optical modes^29^,^30^.

It is well known that the phase plays a key role in the spatiotemporal evolution of an optical field. The simultaneous manipulation of both polarization and phase of an optical field is of particular interest, as it can provide better insights into vector beams. Caustics, as the most important diffraction catastrophe, are especially useful in analyzing diffracting objects^18^. Caustics have been intensively studied, especially since the experimental generation of the Airy beam, due to its fascinating behaviors and potential applications^32^,^33^,^34^,^35^,^36^,^37^,^38^,^39^,^40^. As an important paradigm of ray optics, caustic beams have been generated and demonstrated for their fascinating behaviors and potential applications such as curve trajectory, self-healing and transverse acceleration^36^,^37^,^38^,^39^,^40^. The spectral caustics have recently been used to study the process of high-harmonic generation and predict locations of enhanced intensity within spectra^41^. The interaction between the vortex (scalar one - phase singularity or vectorial one - polarization singularity) and spatial profiles in an optical field remains a popular topic^9^,^15^,^16^ due to their potential applications and fundamental interest. The impact of a caustic phase on an optical field has been intensively explored to manipulate an optical field^36^,^37^,^38^,^39^,^40^. The properties of a caustic beam such as self-healing, curve trajectory have been theoretically and experimentally demonstrated^36^,^37^,^38^,^39^,^40^. However, most of these works are focused on a scalar field. The study of the effect of a caustic phase on a vector vortex field with inhomogeneous SoP in the field cross-section, especially the manipulation of optical angular momentum in a vector vortex optical field, is still scarce. Nevertheless, tailoring the phase of vector beams remains a challenge due to the lack of a reliable way to modulate the phase of a vector beam while leaving its SoP intact.

In this work, an approach to generate vector beams with the desired polarization and phase structures is proposed. We employ a direct phase generating method, resembling a caustic-based approach to accelerating beam synthesis^37^, to generate a cylindrical caustic vector vortex optical field with spatially inhomogeneous SoP in the field cross section. The caustic vector vortex optical field is space-variantly modulated in both phase and SoP in the field cross-section, and it propagates with acceleration trajectories in free space. We present both theoretical and experimental results for the novel properties of the cylindrical caustics vector vortex optical field. The results indicate that the SoP in the field cross-section rotates during propagation due to the existence of the vortex. The distributions of both spin and orbital optical angular momentum fluxes in the field cross-section can be manipulated by the initial vortex, polarization topological charges, and, especially, the caustic phase. This approach provides a novel method to manipulate the distributions of the spin and orbital optical angular momentum fluxes in the field cross-section. Furthermore, understanding their functionality would contribute to applications in the micro-manipulation of particles and atoms.

## Results

### Evolution of distribution of SoP, spin and orbital angular momentum flux density in a caustic vector vortex optical field

We first examine a vector optical field with inhomogeneous linear polarization in a field cross-section as an initial field:


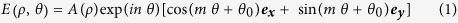


where
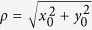
and 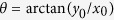 are the polar radius and azimuthal angle in the polar coordinate system, respectively. *m* and *n* are the topological charges of polarization and helical phase, respectively, and *θ*_0_ is the initial phase. ***e***_*x*_ and ***e***_*y*_ are the unit vectors in the x and y-direction, respectively. When *m* = 1, the vector fields describe the radially and azimuthally polarized vector fields for *θ*_0_ = 0 and π/2, respectively. In particular, an appropriate radially dependent phase profile *ψ*(*ρ*) as a function of the polar radius of the initial optical field (which is included in *A*(*ρ*), i.e., *A*(*ρ*) = *A*_0_circ(*ρ*/*r*_0_)exp[-i*ψ*(*ρ*)] in Eq. (1) where *A*_0_ is a constant, circ(.) is the circular function, and *r*_0_ is the radius of the vector field, which is truncated by the SLM) has been adopted to generate the desired caustic with arbitrary convex acceleration trajectories.

Based on the Rayleigh-Sommerfeld diffraction formula^42^, the electric field components (when propagating along the z direction) are expressed as


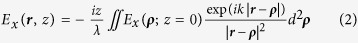



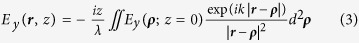



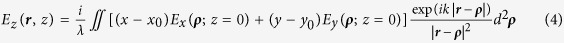
where the polar coordinate is represented by ***ρ*** = *x*_0_***e***_*x*_ + *y*_0_***e***_*y*_ in the initial plane at *z* = 0 and by ***r*** = *x**e***_*x*_ + *y**e***_*y*_ + *z**e***_*z*_ in the plane at a propagation distance *z*. The desired caustic beam with arbitrary acceleration trajectories can be generated by imposing the corresponding spatial phase function *ψ*(*ρ*) onto the initial optical field^18^,^36^,^37^,^38^,^39^,^40^. The experimental setup satisfies the paraxial circumstances, and our full vectorial calculation results confirm that the z-component of field can be neglected compared to the transverse components. Under the paraxial approximation, the transverse energy (TE) flow of the vector cylindrical optical field in the *xy*-plane can be written in the following form:


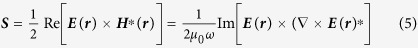


where Re[.] and Im[.] denote the real and imaginary parts, respectively, and the asterisk corresponds to its complex conjugation. *μ*_0_ and *ω* are vacuum permeability and angular frequency, respectively. Therefore, Eq. (5) describes the energy flux distribution at the propagation plane where *z* ≡ constant. The optical angular momentum flux density is^43^


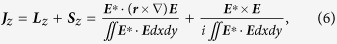


where the first and second terms represent the distributions of the orbital angular momentum flux density ***L***_z_ and the spin angular momentum flux density***S***_z_, respectively.

**Figure 1:**
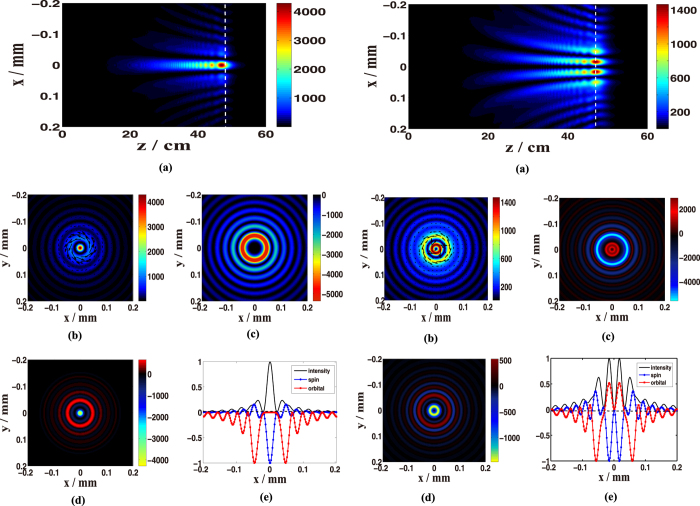
The distributions of a caustic vector vortex optical field at propagation distance *z* = 47 cm (the peak intensity position) with *n* = 2, *m* = 2 (left) and *n* = 2, *m* = 3 (right). The applied caustic phase *ψ*(*ρ*) = 0.1*kρ*^3/2^. (**a**) Intensity distribution in the central longitudinal cross-section with varying propagation distance. (**b**) The intensity distribution and transverse energy flow. Arrows represent the directions of the energy flow. **(c**) The orbital angular momentum flux distribution. (**d**) The spin angular momentum flux distribution. (**e**) Normalized intensity distribution, orbital angular momentum flux distribution, and spin angular momentum flux distribution in the cross-section.

Figure 1 shows the distributions of a caustic vector vortex optical field at propagation distance *z* =47 cm (the peak intensity position) with *n* = 2 and *m* = 2, and with *n* = 2 and *m* = 3. Here, the applied spatial phase function for the curve caustic is set as *ψ*(*ρ*) = 0.1*kρ*^3/2^ and *A*(*ρ*) = *A*_0_exp(-*iψ*(*ρ*)) where *A*_0_ is a constant. The optical field propagates in the curve trajectory during propagation, and the optical field is initially auto-focusing before approaching a peak intensity. The intensity remains at the appropriate peak for a few centimeters, and then it attenuates quickly after the foci, due to the effect of the caustic phase *ψ*(*ρ*) = 0.1*kρ*^3/2^ as showed in Fig. 1(a). The numerical results indicate that the transverse energy flow distribution varies in the field cross-section, as shown in the (b) plots in Fig. 1. The direction of the energy flows in the field cross-section for the case *n* = 2 and *m* = 2 are anticlockwise (see the left plot (b) in Fig. 1), implying that the orbital angular momentum flux density distributions have negative values as shown in Fig. 1(c) (left). On the other hand, for the case with *n* = 2 and *m* = 3, the direction of energy flows in outer rings is anticlockwise, whereas it is clockwise in the inner ring (see the right plot (b) in Fig. 1). This indicates that the orbital angular momentum flux density distributions have opposite values (positive or negative), as shown in Fig 1(c). The spin angular momentum flux density distributions also exhibit opposite values (positive for left-hand circular polarization and negative for right-hand) at different positions in the field cross-section, as shown in plots (d) in Fig. 1. For direct comparison of these distributions in the field cross-section, the corresponding normalized intensity distribution, orbital angular momentum flux distribution and spin angular momentum flux distribution in the cross-section are depicted in Fig. 1(e). Here, all values have been normalized to their peak values.

**Figure 2:**
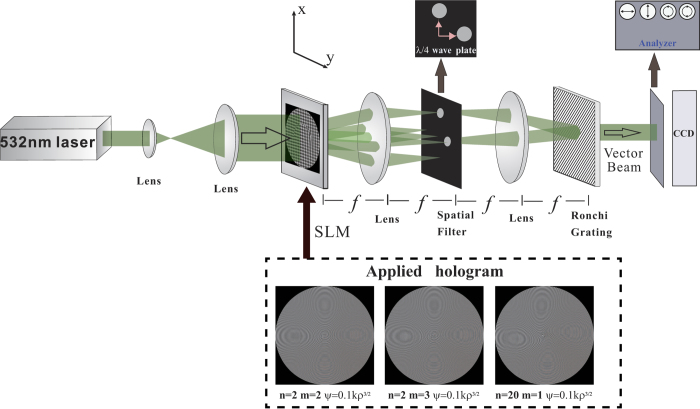
The experimental arrangement for the generation and measurement of a caustic vector vortex optical field. An expanded and collimated laser beam is incident on the Spatial Light Modulator (SLM) with a two-dimensional (2D) holographic grating displayed on the SLM. At the Fourier plane of the 4f system, only two of the first orders, located respectively in the x and y axes, are allowed to pass through two separate open apertures and then converted by two *λ*/4 plates into the left- and right-hand circularly polarized beams, respectively. A Ronchi phase grating is placed in its rear focal plane by 45° with respect to the x direction to recombine the selected orders. The resulting intensity distribution of the beams is recorded by a charge-coupled device (CCD) camera after passing the x-direction linear polarizer, y-direction linear polarizer, left-hand circular polarizer and right-hand circular polarizer analyzers.

**Figure 3:**
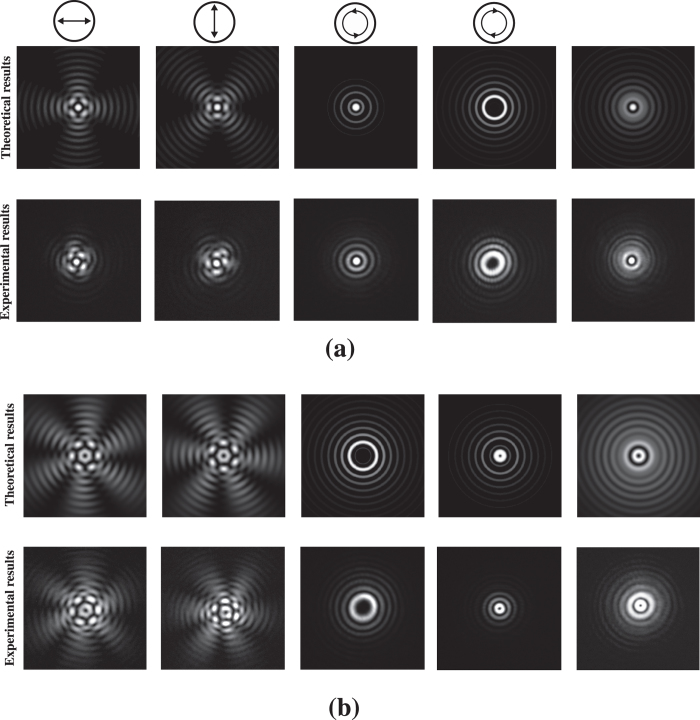
The intensity distributions of a caustic vector vortex optical field after passing or not passing through analyzers at propagation distance *z* = 47 cm with (**a**) *n* = 2 and *m* = 2 or (b) *n* = 2 and *m* = 3. The applied caustic phase *ψ*(*ρ*) = 0.1*kρ*^3/2^. In (a) and (**b**), the upper rows contain theoretical results, and lower rows contain CCD camera images. The corresponding analyzers for the measurements of different polarization components in Fig. 3(a), (b) from columns 1 to 5 are: x-direction linear polarizer; y-direction linear polarizer; left-hand circular polarizer; right-hand circular polarizer; and no polarizer.

The experimental setup for the generation and measurement of a caustic vector vortex optical field is depicted in Fig. 2. The polarization components are measured by the images recorded in the CCD camera after the corresponding analyzers, i.e., x-linear, y-linear, left-hand circular, or right-hand circular polarization analyzers, as shown in Fig. 2. The experimental observations confirm the theoretical predictions for the corresponding intensity distributions of different polarization components in the vector vortex optical field at a propagation distance of *z* = 47 cm, as shown in Fig. 3(a) for the case with *n* = 2 and *m* = 2, and *n* = 2 and *m* = 3. The applied caustic phase is chosen as *ψ*(*ρ*) = 0.1*kρ*^3/2^. In addition, we have applied other caustic phases such as *ψ*(*ρ*) = 0.1*kρ*^7/4^ and *ψ*(*ρ*) = 0.1*kρ*^9/5^ to generate the caustic vector vortex field. Our results show that the physical phenomena are similar to the case with *ψ*(*ρ*) = 0.1*kρ*^3/2^ except for the auto-focus distances, intensities and sizes of the rings at the periphery. The underlying physics for the impact of different caustic phases on a vector vortex optical field are the same as different caustic phases on a scalar field^36^,^37^. These experimental and theoretical results represent different arrangements of SoP in the field cross-section during propagation. The results clearly indicate that the spatial distribution of SoP in the field cross-section can be dynamically manipulated during propagation by choosing the initial vortex and polarization topological charges, as well as the applied caustic phase. These results also confirm that the structural distributions of spin and orbital angular momentum fluxes can be tuned by the initial vortex, polarization topological charges, and the caustic phase.

### SoP rotation in a caustic vector vortex optical field

**Figure 4:**
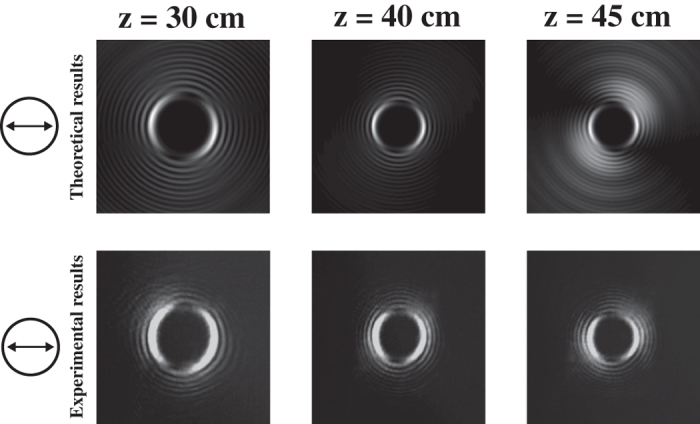
The rotation of x-direction intensity distributions of a caustic vector vortex optical field with *n* = 20, *m* = 1 and *ψ*(*ρ*) = 0.1*kρ*^3/2^ at different propagation distances passing through a linear polarizer. Upper row: theoretical results. Lower row: CCD camera images.

Many studies have demonstrated that a vortex optical field can rotate a micro particle, an atom or even an optical filament. However, the SoP rotation in a vector vortex optical field is still not well understood. Our results indicate that the SoP in the field cross-section can rotate due to the existence of a vortex optical field, as shown in Fig. 4. Here, we use a caustic approach to validate the experimental observations of SoP rotation in the field cross-section at a certain propagation distance. The intensity distribution of the polarization component in the x-direction of a vector vortex optical field with *n* = 20 and *m* = 1 is experimentally observed at different propagation distances using an *x*-linear polarization analyzer. Both the theoretical and experimental results show the rotation of the polarization component during propagation in free space, as shown in Fig. 4.

## Discussion

### Manipulating the arrangement of SoP, spin and orbital angular momentum flux density distributions in the cross-section of a caustic vector vortex optical field

In order to gain further insight on the manipulation of spin and orbital angular momentum flux density distributions, as well as SoP rotation in a caustic vector vortex optical field, we perform a simple theoretical analysis as follows. A vector vortex optical field can be rewritten as:
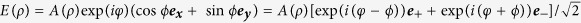
where 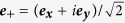 and 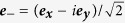 are the unit vectors for left- or right-hand circular polarization, respectively. This means that the vector vortex optical field can be regarded as the superimposition of two vortex fields with opposite circular polarizations. The desired target caustic with arbitrary acceleration trajectories can be generated by imposing the corresponding spatial phase function ψ(*ρ*) on the initial optical field. When a vector vortex optical field is modulated by a caustic phase, the two vortices separate in acceleration trajectories during propagation. They appear in different positions of the field cross-section due to the diffraction effect with different vortex charges. Essentially, the diffraction effect of the same caustic phase on different vortices is different. The coherent superposition of the two caustics vortices with left- and right-hand circular polarization in different positions of the field cross-section leads to the desired spatial distributions of SoP, as well as the orbital and spin optical angular momentum fluxes in the field cross-section.

### Rotation of SoP in the cross-section of the caustic vector vortex optical field

Another interesting phenomenon is the rotation of SoP in the cross-section of the caustic vector vortex optical field during propagation. It is well-known that the SoP remains invariant during propagation in free space. Our results indicate that the distribution of SoP in the cross-section of a vector vortex optical field rotate during propagation in free space due to the existence of a vortex. The vector vortex optical field can be regarded as a superimposition of two vortex optical fields with opposite circular polarizations, and the two vortices propagate in different curve trajectories due to the impact of the caustic phase (*ψ*(*ρ*) = 0.1*kρ*^3/2^ in this work) and diffraction effect of the vortices. Thus, the different SoPs located at different positions in the field cross-section rotate a different angles with respect to the optical axis during propagation, as shown in Fig. 1.

## Methods

### Experimental generation and observation of a caustic vector vortex optical field

In the present study, the experimental setup is illustrated in Fig. 2^30^. In the experiment, a linearly-polarized TEM_00_ laser beam from a semiconductor laser (λ = 532 nm, Laser Quantum Opus 2) is expanded and truncated to illuminate a computer-controlled spatial light modulator (SLM) with 1920 × 1080 pixels (HoloEye Photonics LETO), and then shifted into a 4*f* system composed of a pair of identical lenses with a focal length of *f*. A computer-generated two-dimensional (2D) holographic grating (HG) for the desired vector vortex with a caustic phase profile is displayed on the SLM, which diffracts the incoming light into various diffraction orders. At the Fourier plane of the 4*f* system, only two of the first orders located respectively in the x and y axes are allowed to pass through a spatial filter (with two separate open apertures) and then converted by two λ/4 plates into the left- and right-hand circularly polarized beams, respectively. The selected orders are recombined by the second lens in its rear focal plane, in which a Ronchi phase grating is placed at 45° with respect to the x direction. The period of the HG is adjusted so that its spatial frequency matches that of the Ronchi grating. The gridlike amplitude transmission function of SLM with an HG written on is: *T*(*x*_0_, *y*_0_) = 0.5 + *γ*[cos(2π*f*_0_*x*_0_ + *α*(*x*_0_, *y*_0_) + *ψ*(*x*_0_, *y*_0_)) + cos(2π*f*_0_*y*_0_ + *β*(*x*_0_, *y*_0_) + *ψ*(*x*_0_, *y*_0_))]/4, where *f*_0_ and *γ* are the spatial frequency and modulation depth of HGs, respectively. After being collinearly recombined behind the Ronchi grating, the output beam can be expressed by the superposition of left- and right-hand circular polarization components with a caustic phase as *E*(*x*_0_, *y*_0_) = *A*_0_[exp(*iα*)(***e***_***x***_ + *i**e***_***y***_) + exp(*iβ*)(***e***_***x***_ – *i**e***_***y***_)] = 2*A*_0_exp(*iϕ*)[cos(*ø*)***e***_***x***_ + sin(*ø*)***e***_***y***_], where *ϕ*(*x*_0_, *y*_0_) *=* [*α*(*x*_0_, *y*_0_) + *β*(*x*_0_, *y*_0_)]/2 – *ψ*(*x*_0_, *y*_0_), *ø* (*x*_0_, *y*_0_) = [*α*(*x*_0_, *y*_0_) – *β*(*x*_0_, *y*_0_)]/2 – *ψ*(*x*_0_, *y*_0_), *α*(*x*_0_, *y*_0_) = *ϕ*(*x*_0_, *y*_0_) + *ø* (*x*_0_, *y*_0_) + *ψ*(*x*_0_, *y*_0_), and *β*(*x*_0_, *y*_0_) = *ϕ*(*x*_0_, *y*_0_) – *ø* (*x*_0_, *y*_0_) + *ψ*(*x*_0_, *y*_0_). *A*_0_ is a constant representing the amplitude distribution. The applied holograms for generating the corresponding caustic vector vortex fields in this work are shown in Fig. 2.

## Additional Information

**How to cite this article**: Chen, R.-P. *et al.* Structured caustic vector vortex optical field: manipulating optical angular momentum flux and polarization rotation. *Sci. Rep.* **5**, 10628; doi: 10.1038/srep10628 (2015).
